# Plasma concentration of fucosyltransferase 7 is not associated with the number of clinically overt vaso-occlusive events in sickle cell disease

**DOI:** 10.4314/gmj.v57i3.6

**Published:** 2023-09

**Authors:** Iheanyi E Okpala, Onyinye E Eze, Chinedu A Ezekekwu, Ifeyinwa D Nnakenyi, Gladys U Ilechukwu, Chinedu O Akpa, Emmanuel I Nwani, Collins J Maduka, Ikechukwu O Anigbogu, Ebele D Muoghalu, Ngozi I Ugwu, Ifeoma C Ajuba, John C Aneke, Helen C Okoye, Charles C Nonyelu, Augustine N Duru

**Affiliations:** 1 Department of Haematology and Immunology, University of Nigeria Teaching Hospital, Enugu, Nigeria; 2 Department of Haematology, Enugu State University Teaching Hospital, Enugu, Nigeria; 3 Department of Chemical Pathology, University of Nigeria Teaching Hospital, Enugu, Nigeria; 4 Department of Haematology and Blood Transfusion, Alex Ekwueme Federal University Teaching Hospital, Abakaliki, Nigeria; 5 Department of Haematology, Federal Medical Centre, Umuahia, Nigeria; 6 Haematology Department, Nnamdi Azikiwe University Teaching Hospital, Nnewi, Nigeria

**Keywords:** Fucosyltransferase7, sickle cell disease, adhesion molecules

## Abstract

**Objective:**

To determine if the number of vaso-occlusive events in SCD relates to plasma concentration of fucosyltransferase 7 (FUT7), which catalyses the synthesis of selectin ligands.

**Design:**

A prospective, analytical study.

**Setting:**

Haematology and Chemical Pathology Departments of tertiary healthcare centres

**Participants:**

Steady state HbSS individuals aged 13-45 years, 20 had 3 or more vaso-occlusive crises that required hospital admission in the previous year (with or without complications of SCD); 17 other HbSS persons had 0-1 vaso-occlusive crisis that required hospital admission in the previous year and no disease complications.

**Intervention:**

Steady-state plasma concentrations of FUT7 measured by ELISA were compared between SCD patients who had one vaso-occlusive crisis requiring hospital treatment in the previous year but no disease complications and those who had >3 crises with or without complications.

**Main Outcome Measures:**

Plasma level of FUT7and the number of vaso-occlusive events in each HbSS patient

**Results:**

Mean + standard deviation plasma concentration of FUT7 was 8.6 + 2.7 ng/ml in patients with >3 vasoocclusive crises in the previous year and 7.3 + 1.7 ng/ml in those with 0-1 crisis and no complications; independent sample t-test, p > 0.05, not significantly different.

**Conclusion:**

Plasma concentration of fucosyltransferase7 is not associated with the number of vaso-occlusive events in sickle cell disease.

**Funding:**

None declared

## Introduction

Homozygous (HbSS) sickle cell disease (SCD) is inherited and characterised by the variant haemoglobin S in erythrocytes and clinical illness due to this abnormal blood pigment.^1^-^4^ With an estimated 30 million people affected worldwide, SCD is humanity's most common inherited blood condition.^5^,^6^ The mechanisms of SCD include erythrocyte sickling, chronic haemolysis, susceptibility to infections, and occlusion of blood vessels, leading to ischemic injury to various tissues.^3^,^4^ Vaso-occlusion in SCD results from the adherence of erythrocytes, leucocytes and platelets to each other and the vascular endothelium.^7^-^12^ This cell adherence is mediated by cell surface adhesion molecules, including selectins and their ligands (receptors), such as platelet-selectin glycoprotein ligand-1 (PSGL-1 or CD162).^7^-^12^

Notwithstanding its name, platelet-selectin glycoprotein ligand-1 binds to all the three selectins that occur naturally in humans: selectin L (CD62L) expressed exclusively in leukocytes, selectin P (CD62P) found in platelets and vascular endothelial cells, and selectin E (CD62E) or endothelial selectin expressed in vascular endothelium.^13^ Selectin P on vascular endothelial cells contributes to the adhesion of sickle erythrocytes to the endothelium.^10^ Therefore, selectin P can mediate the adhesion of both erythrocytes and leucocytes to vascular endothelium and adherence of platelets to leucocytes. Since hetero-cellular aggregates of blood cells and their adherence to vascular endothelium contribute to vaso-occlusion in SCD, the selectins and their naturally occurring ligands are crucial in the pathogenesis of ischemic tissue injury characteristic of this disorder. Selectins and their ligands mediate the initial phase (rolling) of leucocyte adhesion to vascular endothelium.^14^,^15^ They are more important for mediating the rolling and the recruitment of blood cells to the vascular wall rather than firm adhesion.^14^,^15^ Increased expression of selectin L on leucocytes is associated with vaso-occlusive crisis and complications in SCD, such as stroke, nephropathy and avascular necrosis of the head of the femur.^16^ Selectin P, in particular, has a strategic role in SCD-related vaso-occlusion because it is the one adhesion molecule which is involved in the adhesion of all three types of blood cells (erythrocytes, leucocytes and platelets) not only to each other but also to the blood vessel endothelium.^10^,^13^ The clinical importance of selectin P and its ligands to vaso-occlusion in SCD is underlined by the fact that the only licensed monoclonal antibody, and the other currently undergoing clinical trial for the treatment of this disorder, are antiselectin P antibodies.^17^,^18^

Clinical experience and research show that marked individual variations occur in the phenotype of SCD.^19^ So, whereas some affected persons live relatively normal lives without disease complications and are seldom in crisis, others suffer from frequent vaso-occlusive crises (painful episodes) requiring hospital admissions. A persisting challenge of SCD is understanding the biological basis of individual differences in clinical phenotype and applying this knowledge to patient care. Information from several studies indicates that the degree of illness or disease phenotype in each HbSS individual is the net effect of various genetic and environmental variables acting in concert with the abnormal haemoglobin S.^19^ It has been demonstrated that selectins and their ligands contribute to vaso-occlusion in SCD.^7^-^12^ Previous studies revealed that increased expression of selectin on leucocytes is associated with vaso-occlusive crisis and complications of this inherited disorder.^16^ Extending these investigations revealed no association between single nucleotide polymorphisms in the selectin L gene (*SELL*) and vaso-occlusive complications of SCD.^20^ Although the understanding of the clinical importance of selectins and their ligands in SCD has since improved,^17^,^18^, ^21^-^23^ there is still little information on whether the number of vaso-occlusive events in SCD relates with plasma levels of the biosynthetic enzymes for selectin ligands. This study aimed to find out if the number of clinically overt vasoocclusive events in SCD is associated with plasma concentration of fucosyltransferase 7 (FUT7), which catalyses the synthesis of sialyl Lewis x (sLe^x^) tetrasaccharide, the core carbohydrate moiety in some ligands for human selectins.^24^-^26^

## Methods

### Study Population

This study was given the University of Nigeria Teaching Hospital Health Research Ethics Committee approval UNTH/HREC/2022/04/3 on 16^th^ April 2022. Following informed consent and assent as appropriate, two groups of homozygous (HbSS) SCD patients in steady state were enrolled. None of the patients was on hydroxyurea (hydroxycarbamide) therapy. Each individual was of age 13 years or greater, and the HbSS phenotype had been confirmed by high-performance liquid chromatography (HPLC). Group I was made up of HbSS individuals who had 3 or more episodes of vaso-occlusive crises that required hospital treatment in the previous year and who may or may not have developed any vaso-occlusive complication of SCD (such as stroke, avascular necrosis of the femoral head, sickle nephropathy and acute chest syndrome). Group II included HbSS persons who had 0-1 vaso-occlusive crisis that required hospital treatment in the previous year and had not developed any vaso-occlusive complication of SCD. The exclusion criteria were age over 70 years, blood transfusion in the previous 4 months, treatment with hydroxycarbamide (hydroxyurea), regular blood transfusion program or other agents which may affect the frequency of vaso-occlusive crisis, co-existing chronic disorders unrelated to SCD; e.g. asthma and diabetes mellitus.

### Sample Size

The minimum sample size in each study group was calculated from the formula^27^

N = (Z1- X)^2^ (P) (1-P)/D^2^, where N is the minimum sample size at 95% confidence level: Z1 - X= 1.96 is the proportion of the normal distribution curve within the mean + 2SD; P is the prevalence of the condition to be studied. The prevalence of HbSS phenotype in Nigeria is 0.01^1^.

If the power of the study indicated as D is set at 0.05, N = (1.96)^2^ x (0.01) x (0.99) divided by (0.05)^2^ = 15.2. So, a minimum of 16 HbSS persons were required in each group.

### Determination of Plasma Concentration of FUT7 and Blood Cell Counts

The concentration of FUT7 in each participant's plasma sample was measured using a commercially available ELISA kit with Lot Number 20210507C from MyBiosource Inc, USA, following the manufacturer's instructions. The blood haemoglobin concentration, leucocyte and platelet counts were determined with the “Mythic” automated blood cell counter.

### Data Analyses

Data generated were analysed using the Statistical Package for Social Sciences (SPSS version 23, IBM, Armonk, NY, USA). Plasma FUT7 levels in Groups I and II were compared using the two-tailed independent sample t-test and the Mann-Whitney U test. A P-value of < 0.05 was taken as indicating a significant difference.

## Results

Thirty-seven HbSS patients were recruited, 18 males and 19 females aged 13-45 years. Group I included 20 patients with 3 or more vaso-occlusive crises that required hospital admission the previous year, 12 males and 8 females with an age range of 13-41 years and mean age + SD of 22.4 + 6.2 years. Group II included 17 patients with 0-1 vaso-occlusive crisis that required hospital admission in the previous year and no complications of SCD, 6 males and 11 females aged 18-45 years with mean age + SD 24.5 + 8.1 years. The t-test showed no significant difference between the mean + SD FUT7 plasma concentration of 8.6 + 2.7 ng/ml in Group I and 7.3 + 1.7 ng/ml in Group II; p > 0.05. Mann-Whitney U test showed no significant difference in plasma FUT7 between the two groups; U = 123, p = 0.15. Demography data, SCD phenotype and plasma FUT7 concentration for all study participants are provided in Table 1.

**Table 1:** Disease phenotype and plasma levels of fucosyl-transferase7 in steady-state HbSS patients

Group 1: >3 VOC/yr with or without complications				
ID	Age/Sex	Crisis	NOC	FUT7 ng/ml
**1**	16F	3	1	7.8
**2**	20M	4	1	8
**3**	18M	3	2	10.4
**4**	20F	4	0	5
**5**	23F	6	1	6.3
**6**	21M	4	1	5.8
**7**	28M	3	0	5.9
**8**	31M	3	2	13.1
**9**	20F	4	0	7.6
**10**	26F	6	1	7.2
**11**	18M	3	1	7.3
**12**	22F	3	2	12.4
**13**	18M	4	1	15
**14**	41F	4	0	10.9
**15**	28F	3	1	7.5
**16**	13M	7	0	10.3
**17**	18M	5	1	5.5
**18**	24M	4	2	8.1
**19**	22M	3	0	8.5
**20**	20M	4	2	8.3
**Mean**				**8.6 ng/ml**
**SD**				**2.7**

**Group II: 0-1 VOC /yr without complications**				
**ID**	**Age/Sex**	**Crisis**	**NOC**	**FUT7 ng/ml**
**21**	18M	1	0	10.8
**22**	45F	1	0	7.3
**23**	18F	1	0	7
**24**	32F	1	0	8.8
**25**	23M	1	0	5.6
**26**	19F	1	0	6.9
**27**	27M	1	0	7.3
**28**	18M	1	0	6.9
**29**	19M	1	0	8.4
**30**	21F	1	0	7.4
**31**	18F	1	0	6.3
**32**	23F	1	0	5.2
**33**	37F	1	0	7.8
**34**	25F	1	0	10.6
**35**	23F	1	0	7.9
**36**	16M	1	0	4.8
**37**	34F	1	0	5.5
**Mean**				**7.3 ng/ml**
**SD**				**1.7**

t = 1.7 at degree of freedom 35, not significant

Crisis: Number of vaso-occlusive crisis in the previous year; NOC: Number of vaso-occlusive complications of sickle cell disease; M: Male; F: Female; SD: standard deviation.

The blood haemoglobin concentration in SCD patients who had >3 crises vaso-occlusive crises in the previous year was significantly lower than in those with 0-1 crisis, p = 0.00, Table 2. The leucocyte and platelet counts in the two groups were comparable.

**Table 2:** Steady state full blood count in HbSS patients

Group 1: >3 VOC/yr with or without complications				
ID	Age/Sex	Hb	WBC	Platelets
**1**	16F	8.2	10.6	284
**2**	20M	6.9	10.4	191
**3**	18M	7.2	10.1	407
**4**	20F	6.3	6.2	236
**5**	23F	5.7	11.2	354
**6**	21M	7.7	11.0	423
**7**	28M	6	3.5	224
**8**	31M	6	7.0	501
**9**	20F	5.9	10.4	159
**10**	26F	6.3	6.2	236
**11**	18M	7.2	10.1	407
**12**	22F	6.2	9.5	355
**13**	18M	7.0	11.5	413
**14**	41F	6.3	11.2	411
**15**	28F	6.7	4.3	248
**16**	13M	5.9	7.6	400
**17**	18M	6.8	10.7	221
**18**	24M	7.1	9.7	341
**19**	22M	6.0	6.4	298
**20**	20M	6.6	5.8	302
**Mean**		**6.6**	**8.6**	**320**
**SD**		**0.7**	**2.5**	**93.9**
**t**		**-7.5**	**1.8**	**1.3**
**P**		**0.00 (sig)**	**0.8 (ns)**	**0.2 (ns)**

**Group II: 0-1 VOC /yr without complications**				
**ID**	**Age/Sex**	**Hb**	**WBC**	**Platelets**
**21**	18M	9.3	6.6	265
**22**	45F	7.6	7.3	406
**23**	18F	7.1	8.2	350
**24**	32F	8.5	8.6	211
**25**	23M	9.2	9.3	358
**26**	19F	7.3	9.2	400
**27**	27M	9.0	8.1	302
**28**	18M	9.8	7.9	317
**29**	19M	8.8	4.8	111
**30**	21F	8.1	8.2	162
**31**	18F	9.9	5.6	281
**32**	23F	7.6	7.4	243
**33**	37F	8.9	8.1	251
**34**	25F	7.7	6.3	217
**35**	23F	8.4	7.8	328
**36**	16M	9.1	7.2	288
**37**	34F	8.0	6.4	300
**Mean**		**8.5**	**7.5**	**281**
**SD**		**0.86**	**1.2**	**7.8**
**t**		**-7.5**	**1.8**	**1.3**
**p**		**0.00 (sig)**	**0.8 (ns)**	**0.2 (ns)**

Hb: Haemoglobin concentration (g/dl), WBC: White Blood Cell Count (x10^9^/L), Platelets: Platelet Count (x10^9^/L) M: Male, F: Female, VOC: Vaso-Occlusive Crisis, SD: Standard Deviation, sig: significant, ns: not significant

## Discussion

The findings from this study suggest that the number of clinically overt vaso-occlusive events in SCD is not associated with plasma concentration of fucosyltransferase7, the enzyme that catalyses the biosynthesis of a core carbohydrate component (sLe^x^) in naturally occurring ring ligands of human selectins. These ligands include PSGL-1, the hemopoietic stem cell marker CD34, glycosylation-dependent cell adhesion molecule 1 (GlyCAM1) and mucosal addressin cell adhesion molecule 1 (MAd-CAM1), all of which contain a sulphated form of the sLe^x^ tetrasaccharide.^28^-^38^ However, there are other selectin ligands that occur naturally in humans which do not contain sLe^x^, the similar tetrasaccharide sLe^a^, nor structurally related carbohydrate moieties. For example, heparin and heparan sulphate do not contain carbohydrate groups structurally related to sLe^x^ or sLe^a^. However, they can bind to selectins L and P.^32^ It is plausible that the presence in the body of selectin ligands that do not contain sLe^x^, the synthesis of which is independent of FUT7, might enable blood and vascular endothelial cells to adhere to each other and cause vaso-occlusion in SCD. So, the number of vaso-occlusive crises and the development of complications in SCD might not have a statistically significant relationship with the plasma concentration of FUT7, as was observed in this study.

Consistent with this concept, findings from other studies suggest that fucosyltransferase 4, which, like FUT7, is also an alpha (1,3) fucosyltransferase, contributes to selectin-dependent leukocyte homing.^24^,^25^,^33^ This process involves intercellular adhesion as it occurs in SCD-related vaso-occlusion.^24^,^25^ Mice deficient in FUT7 retained the ability to recruit leukocytes to sites of inflammation but could not do so when the genes for FUT4 were knocked out.^33^ It is noteworthy that FUT7 and FUT4 each show distinct acceptor specificities. Whereas FUT7 adds fucose residues to 2,3-sialylated lactosamine acceptors to form sLe^x^, FUT4 catalyses the addition of fucose to *non*-sialylated lactosamine units to form Lewis x (Le^x^).^34^,^35^ There is evidence that the molecular environment or context within which the carbohydrate moiety in a selectin ligand exists (the surrounding atoms or molecules) affects its affinity for a specific selectin.^35^,^36^ Whereas selectin P, for example, binds best to PSGL-1, which contains sLe^x^, it also has a high affinity for non-sialylated glycosphingolipids, CD24 and sulphatides.^36^,^37^ Also, selectin L binds preferentially to glycoproteins. It also has an affinity for heparin and heparan sulphate.^32^ Furthermore, the human ligands for selectin E include those that contain sLex and the glycosphingolipid VIM2 epitope.^26^ Taken together, the structural differences observed among the naturally-occurring ligands for human selectins support the concept that while those containing sLe^x^ synthesised by FUT7 are important, they are not the only counter receptors that can function in selectin-mediated cell adhesion, such as occurs during blood vessel occlusion in SCD.

## Conclusion

Although fucosyltransferase7 is important in the biosynthesis of a core carbohydrate moiety in human selectin ligands involved in SCD-related vaso-occlusion, the plasma concentration of this enzyme does not significantly associate with the number of clinically overt vasoocclusive events in this inherited globin disorder.
